# Case report: Von Hippel-Lindau syndrome with multisystem involvement: a therapeutic dilemma

**DOI:** 10.3389/fonc.2025.1633911

**Published:** 2025-10-14

**Authors:** Shuai He, Cong Wang, Jing Yang, Juan Shen, Xiaozhu Zeng, Jun Zheng, Yongquan Wang

**Affiliations:** ^1^ Department of Urology, The First Affiliated Hospital (Southwest Hospital) of Army Medical University, Chongqing, China; ^2^ Institute of Urology, The First Affiliated Hospital (Southwest Hospital) of Army Medical University, Chongqing, China; ^3^ International Center for Aging and Cancer, Hainan Medical University, Haikou, Hainan, China; ^4^ Department of Oncology, Cancer Prevention and Treatment Institute of Chengdu, Chengdu Fifth People’s Hospital, Chengdu, Sichuan, China

**Keywords:** von Hippel-Lindau syndrome, case report, aortic dissection, multidisciplinary management, early recognition

## Abstract

We present a rare case of von Hippel-Lindau (VHL) syndrome type 2B, characterized by multisystem involvement including Stanford type B aortic dissection, pheochromocytoma, renal cell carcinoma (RCC), cerebellar hemangioblastoma with obstructive hydrocephalus, and extensive visceral cysts. This case highlights critical therapeutic dilemmas: urgent aortic repair versus risks of catecholamine surge from pheochromocytoma resection, and neurosurgical hazards of cerebellar lesions. We describe the diagnostic and therapeutic challenges associated with VHL syndrome, culminating in the development of a preliminary treatment strategy following multidisciplinary team (MDT) discussions. This study underscores the critical importance of early recognition and systematic MDT management to optimize clinical outcomes in VHL syndrome.

## Introduction

Von Hippel-Lindau (VHL) syndrome is an autosomal dominant disorder caused by mutations in the VHL tumor suppressor gene, characterized by visceral cysts and neoplasms including pheochromocytomas, renal cell carcinoma (RCC), pancreatic tumors, and central nervous system (CNS) hemangioblastomas, etc (1–3). VHL syndrome is classified into distinct clinical subtypes (Type 1, 2A, 2B, and 2C) based on whether specific neoplasms (such as pheochromocytoma and RCC) are present or absent (2, 4). Globally, this autosomal dominant disorder affects approximately 1 in 36,000 individuals (5, 6), yet its diagnosis is frequently delayed due to nonspecific initial manifestations and variable penetrance (7–9). The multisystem involvement and complexity of VHL-related lesions pose significant therapeutic challenges.

Pheochromocytoma, a hallmark of VHL syndrome, results from excessive catecholamine secretion. This typically causes paroxysmal or sustained hypertension by overstimulating adrenergic receptors. Hypertension in this context represents a secondary etiology, distinct from primary (essential) hypertension, with its pathogenesis directly linked to tumor-derived catecholamine excess. Chronic exposure to elevated catecholamines induces systemic hemodynamic disturbances, including persistent vasoconstriction and endothelial dysfunction, which collectively contribute to end-organ damage. Notably, sustained hypertension may precipitate catastrophic vascular complications such as aortic dissection—a life-threatening condition characterized by intimal tear and false lumen formation. Despite the well-established role of hypertension in aortic wall degeneration, aortic dissection remains exceptionally rare in VHL syndrome.

Herein, we present a unique case of VHL syndrome type 2B manifesting with multisystem involvement—Stanford type B aortic dissection, pheochromocytoma, RCC, multiple cysts, and cerebellar hemangioblastoma with obstructive hydrocephalus. This case vividly illustrates the severe therapeutic challenges and critical decision-making points in managing such complex presentations.

## Case presentation

**Figure 1:**
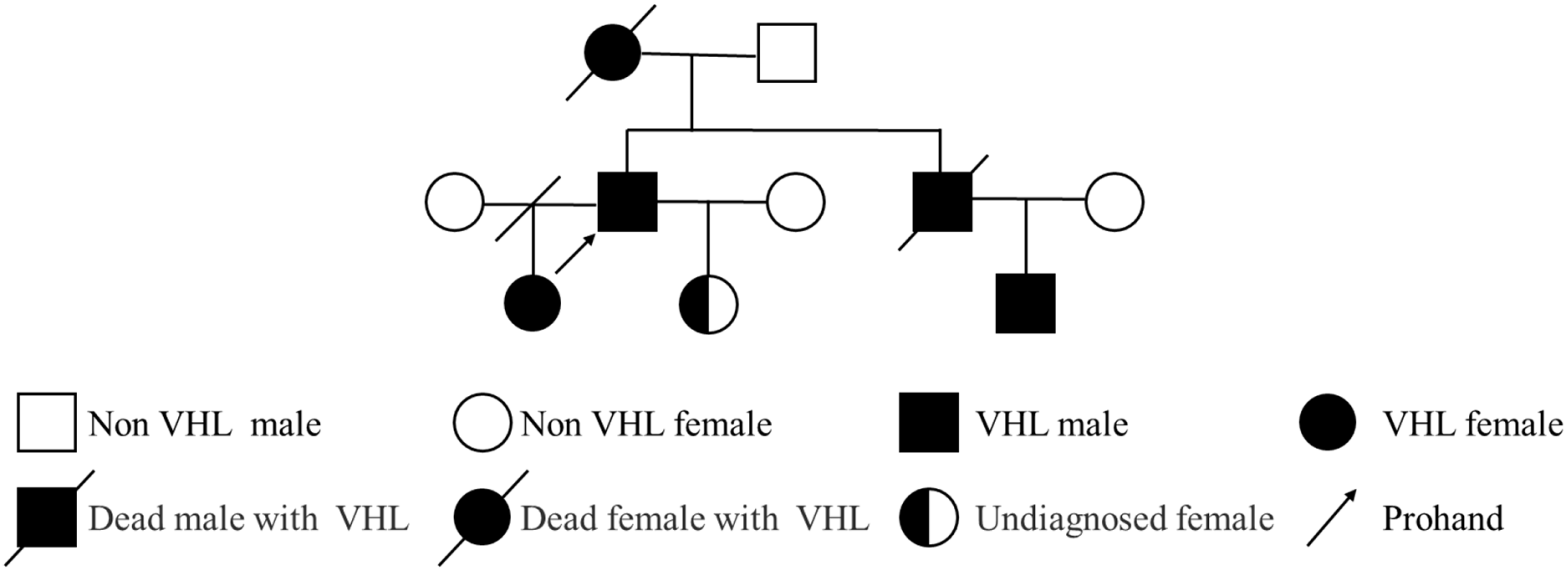
Pedigree diagram of the patient with VHL syndrome. The pedigree illustrates three generations (10 individuals) with VHL syndrome. Symbols: squares represent males; circles represent females; filled symbols denote affected individuals; half-filled symbols represent individuals with unconfirmed status; slashes (/) through symbols indicate deceased individuals; slash through the connecting line indicates divorce; the arrow points to the proband.

We describe a 50-year-old male (Height: 170 cm, Weight: 65 kg, BMI: 22.49 kg/m²) with a family history of VHL syndrome (Family pedigree illustrated in **Figure 1**).

The patient’s medical history began over a decade ago when he initially presented with dizziness and was diagnosed with hypertension (peak blood pressure: 170/100 mmHg) at a local hospital. The initial diagnostic workup was limited to confirming elevated blood pressure and lacked a standardized assessment for secondary causes. Specifically, no biochemical screening for catecholamine excess or dedicated adrenal imaging was performed, leading to a delay in diagnosing the underlying pheochromocytoma by more than ten years. Despite this diagnosis, the patient demonstrated poor adherence to the prescribed antihypertensive therapy, likely due to insufficient awareness of his condition’s severity. Four years prior to admission, surveillance imaging incidentally identified a left adrenal nodule (1.0×1.0 cm) and a right renal lesion (1.0×1.0 cm), yet no further diagnostic evaluation or intervention was initiated. Notably, one month before presentation, routine surveillance revealed a Stanford type B (DeBakey III) aortic dissection, prompting urgent endovascular aortic stent graft placement at another institution. The decision for emergent endovascular repair was made by the vascular surgery team at the referring hospital in accordance with standard aortic dissection management guidelines.

**Figure 2:**
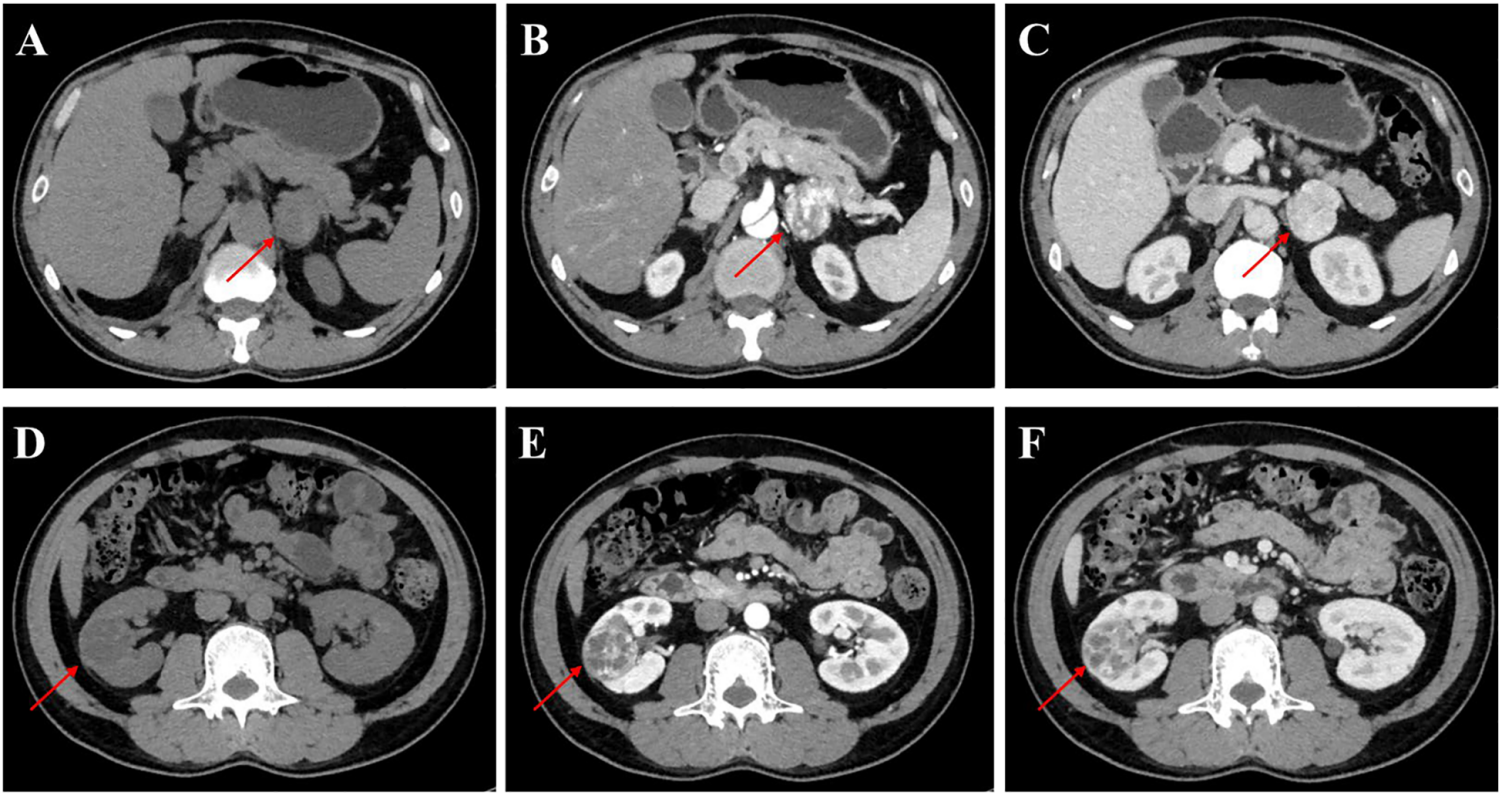
Contrast-enhanced CT imaging of the left adrenal gland and kidney in the patient. **(A-C)** Axial CT images of the left adrenal gland: **(A)** Non-contrast phase demonstrating a well-circumscribed mass (arrow) measuring 4.1×3.5 cm, **(B)** Arterial phase (AP) showing heterogeneous enhancement of the adrenal lesion, **(C)** Venous phase (VP) with persistent contrast retention in the mass. **(D-F)** Coronal CT images of the left kidney: **(D)** Unenhanced scan revealing a 3.5×3.5 cm hypodense renal mass (arrowhead), **(E)** AP highlighting peripheral nodular enhancement, **(F)** VP demonstrating washout pattern characteristic of RCC.

**Figure 3:**
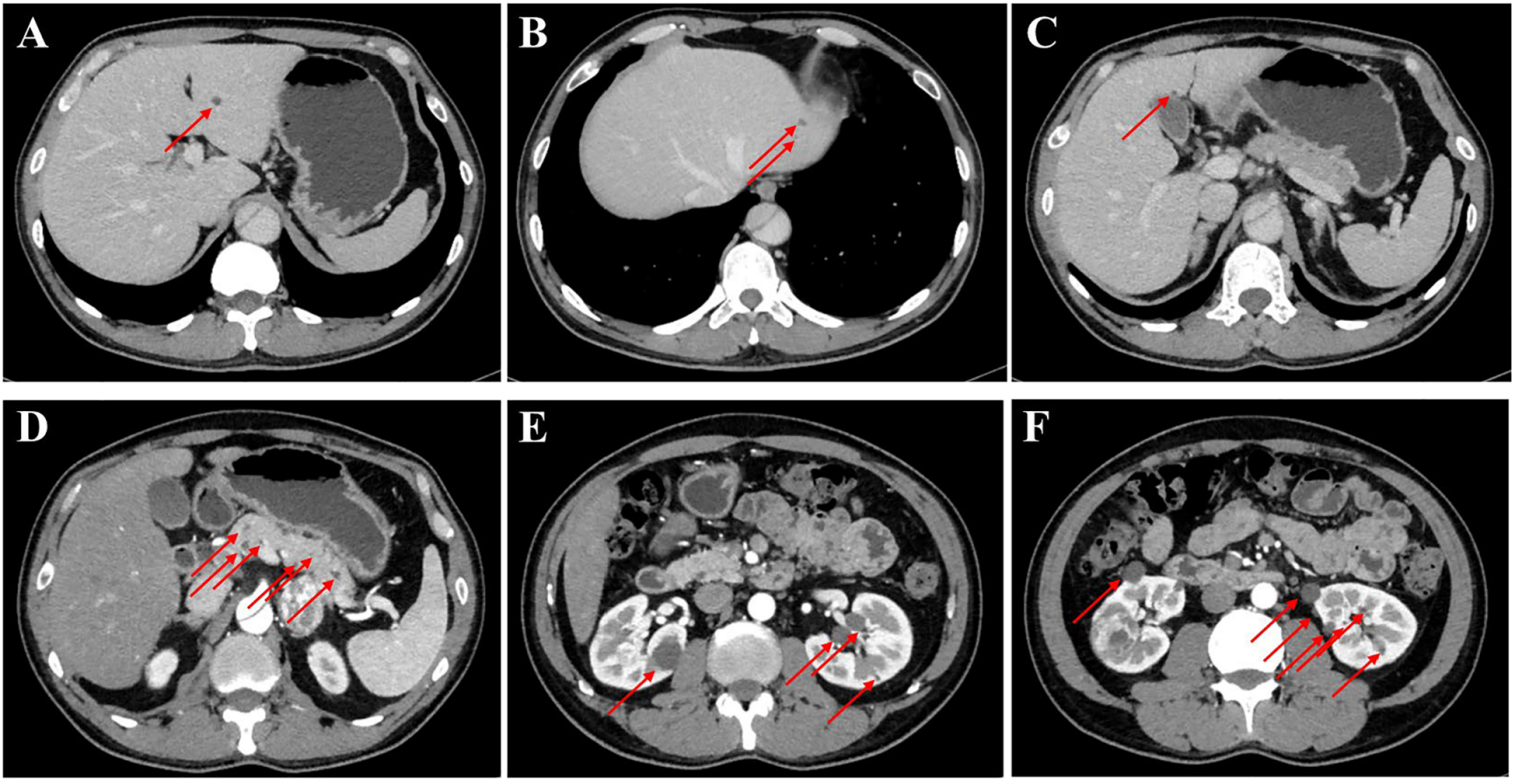
Contrast-enhanced CT imaging of the upper abdomen in the patient. **(A–C)** Axial contrast-enhanced CT images of the liver and the arrow indicates the mass on the liver. **(D)** The contrast-enhanced CT image of the pancreas, revealing that the arrow indicates the mass on the pancreas. **(E, F)** The contrast-enhanced CT images of bilateral kidneys and arrows explain multiple cysts of both kidneys.

The patient was admitted to our department for further management of progressively enlarging left adrenal and right renal masses. A recent contrast-enhanced abdominal CT scan demonstrated substantial growth of the lesions, with the left adrenal mass measuring 4.1×3.5 cm and the right renal mass 3.5×3.5 cm (**Figure 2**). Upon adjusting the window width during image analysis, an additional renal mass measuring 1.3 × 1.4 cm was identified in the right kidney (**Supplementary Figure 1**). The imaging characteristics were consistent with pheochromocytoma and RCC. Subsequent serological evaluation revealed markedly elevated normetanephrine levels (**Supplementary Table 1**), which definitively confirmed the diagnosis of pheochromocytoma. Additionally, multiple hepatic, pancreatic and bilateral renal lesions were observed in the liver, pancreas and bilateral kidneys, radiologically consistent with hepatic hemangiomas and simple pancreas/renal/hepatic cysts (**Figure 3**, the number of renal cysts > 30).

**Figure 4:**
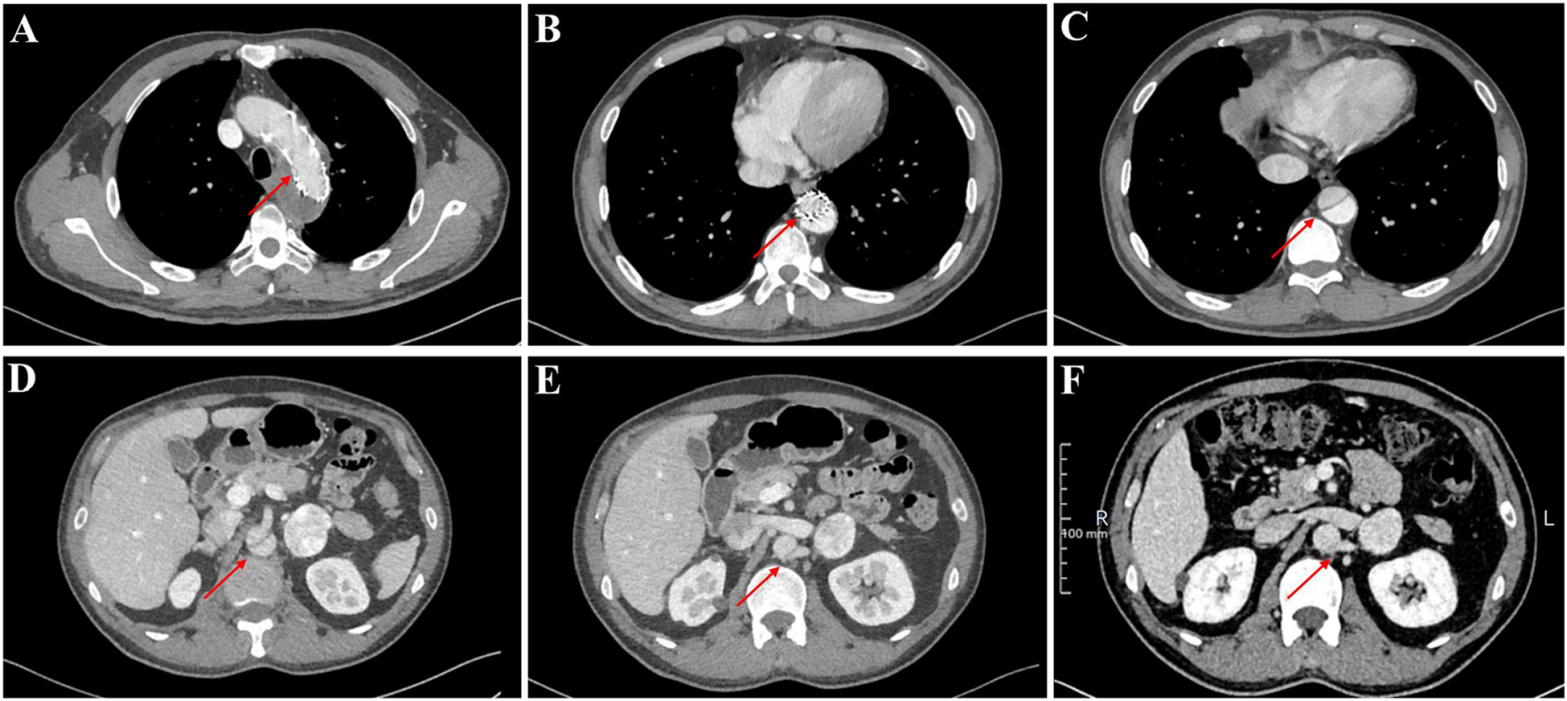
The thoracoabdominal CT angiography (CTA) findings of the patient. **(A–E)** Current CTA study performed at our institution. **(F)** Prior external CTA for comparison.

Notably, our evaluation revealed a persistent aortic dissection extending from the distal descending aorta to the abdominal aorta, raising concerns regarding the efficacy of the prior endovascular stent grafting. To elucidate whether this represented disease progression or incomplete exclusion of the false lumen, we conducted a comparative analysis of pre- and post-procedural CT imaging. The dissection morphology and extent demonstrated no significant interval change, indicating failure of the initial endograft to achieve complete sealing. Further scrutiny of the CT angiography identified the left renal artery originating from the false lumen (**Figure 4**). Therefore, complete endovascular exclusion of the dissection flap would thus risk compromising left renal perfusion, potentially leading to ischemic nephropathy—a therapeutic dilemma corroborated through MDT consultation with the original surgical team.

The diagnosis was established through a combination of imaging and biochemical findings. The adrenal mass displayed classic radiological characteristics of pheochromocytoma, including well-circumscribed borders, heterogeneous enhancement, and persistent contrast retention. The renal mass exhibited enhancement patterns highly suggestive of RCC. The concurrent presence of multiple pancreatic and renal cysts further indicated a systemic disorder. Together, the clinical triad of pheochromocytoma, RCC, and multiple visceral cysts—supported by a significant family history—led to a presumptive diagnosis of VHL syndrome, prompting further multidisciplinary investigations for confirmation and staging.

Prompted MDT investigations included: (1) pedigree analysis revealing an autosomal dominant inheritance pattern (**Figure 1**), with four affected relatives across three generations: maternal death from VHL-associated RCC, a deceased sibling with cerebellar hemangioblastoma, an afflicted nephew with visceral manifestations, and an eldest daughter confirmed with germline VHL mutation under targeted therapy; (2) Brain magnetic resonance imaging (MRI) identifying heterogeneously enhancing lesions in the left cerebellar hemisphere and vermis, radiologically confirmed as cerebellar hemangioblastoma with fourth ventricular obstruction and secondary hydrocephalus (**Supplementary Figure 2**); (3) systematic screening via testicular ultrasonography and advanced ophthalmological imaging (optical coherence tomography (OCT) and ultra-widefield fundus angiography), both yielding unremarkable findings.

The patient’s clinical presentation fulfills the diagnostic criteria for VHL syndrome and aligns with VHL type 2B per the revised classification system (2, 4) (The chronological sequence of the patient’s clinical course is summarized in **Supplementary Figure 3**).

## Discussion

Our case delineates a rare and severe presentation of VHL syndrome type 2B, exceptionally complicated by aortic dissection—a previously unreported association. The concurrence of a catecholamine-secreting tumor, aortic dissection, renal malignancies, and a neurosurgical emergency exemplifies the profound therapeutic dilemma inherent in multisystem VHL disease and underscores the imperative for MDT coordination.

### Therapeutic dilemmas—multisystem pathological conflicts and decision-making paradoxes

This case of VHL syndrome type 2B illustrates the profound complexity of multisystem involvement, encompassing Stanford type B aortic dissection, pheochromocytoma, RCC, cerebellar hemangioblastoma with obstructive hydrocephalus, and extensive visceral cysts. The interaction between these conditions creates a major treatment challenge: conventional treatment approaches (e.g., treating the most urgent condition first) may not work when multiple life-threatening problems occur together. For instance, despite RCC’s malignant potential and association with metastatic mortality, its resection was deferred due to the acute risks posed by concurrent emergencies: (1) untreated aortic dissection (>50% mortality within 48 hours if ruptured (10, 11); (2) cerebellar hemangioblastoma-induced neurological catastrophe (e.g., brainstem compression); and (3) pheochromocytoma-related catecholamine storms precipitating multiorgan failure.

Central to this dilemma is the bidirectional pathophysiology linking pheochromocytoma and aortic dissection. Catecholamine excess directly drove hypertensive aortic degeneration, yet guideline-recommended pheochromocytoma resection introduced critical risks: (1) perioperative hemodynamic instability exacerbating aortic wall stress, and (2) surgical interventions triggering catecholamine surges from untreated pheochromocytoma. This challenge shows that standard treatment plans are insufficient for complex VHL cases. It requires an MDT to create a customized management strategy that addresses the most urgent risks.

### MDT management: stratified intervention and coordinated care

The concurrent management of three acute life-threatening conditions—aortic dissection, cerebellar hemangioblastoma with obstructive hydrocephalus, and pheochromocytoma—constitutes the central challenge and focus of MDT deliberation in this case. While therapeutic prioritization among these emergencies remains contentious, optimizing drug therapy is essential: (1) A β-blocker (e.g., metoprolol) combined with a vasodilator (e.g., sodium nitroprusside) must be initiated immediately to maintain systolic blood pressure (SBP) within 100–120 mmHg and heart rate (HR) <60 bpm, thereby mitigating aortic wall stress; (2) Preemptive α-adrenergic antagonism (e.g., phenoxybenzamine) is indispensable to abrogate the peripheral effects of catecholamine surges. The MDT consensus prioritized aortic dissection stabilization as the initial therapeutic imperative, with particular attention to the left renal artery originating from the false lumen. Adequate preoperative preparation and emergency plans, and the left kidney should be preserved as much as possible. The patient is then treated for central hemangioblastoma and given that the patient does not have CNS symptoms, stereotactic radiotherapy is preferred over surgical resection, which may achieve the treatment of central hemangioblastoma, and avoided surgical stress-induced pheochromocytoma activation (12, 13). Finally, the left pheochromocytoma was treated laparoscopically.

Although the proposed MDT plan could not be implemented due to the patient’s discharge amidst overwhelming medical complexity and financial constraints, our MDT deliberations yielded a structured strategy worth discussing. Had it been executed, the anticipated therapeutic sequence would have involved: (1) continued strict hemodynamic control and monitoring to mitigate aortic stress; (2) stereotactic radiotherapy for the cerebellar hemangioblastoma, aiming to minimize neurological risk while avoiding surgical stimulation of the pheochromocytoma; and (3) subsequent laparoscopic resection of the pheochromocytoma following adequate α- and β-blockade. Even with ideal implementation, several challenges would have persisted, including the need for lifelong surveillance for new VHL-related lesions, the risk of metastatic progression from the RCC, and the possibility of aortic dissection extension or complications despite endovascular repair. This case highlights that in resource-limited settings, even well-designed MDT plans may encounter barriers to implementation, as demonstrated here. Therefore, early diagnosis and intervention—before the onset of multisystem crises—are crucial to improving outcomes in VHL syndrome.

### Broader clinical implications: the imperative for evaluating secondary hypertension

This case underscores a critical lesson in general internal medicine: the necessity of a thorough evaluation for secondary causes in patients with new-onset hypertension. Our patient’s decade-long history of undiagnosed pheochromocytoma-driven hypertension, which ultimately culminated in a catastrophic aortic dissection, serves as a stark reminder. Hypertension, particularly when resistant to conventional therapy, onset at a young age, or accompanied by paroxysmal symptoms (e.g., headache, palpitation, sweating), warrants a systematic workup. This should include biochemical screening for catecholamines to exclude pheochromocytoma. Such an approach could have potentially identified the underlying cause years earlier, allowing for timely intervention and possibly averting life-threatening complications. Therefore, beyond the specific context of VHL, this case advocates for a heightened index of suspicion for secondary hypertension in clinical practice.

### Importance of early diagnosis: complication prevention and genetic risk mitigation

VHL syndrome manifests through diverse multi-organ lesions that present at varying times. Patients often initially exhibit isolated symptoms such as hypertension, retinal hemorrhage, or incidentally detected abdominal masses, often leading to diagnostic delay and underrecognition. Literature documents many cases where VHL was overlooked despite characteristic signs (**Supplementary Table 2**) (7–9), a pattern also seen in our experience: (1) Despite a strong family history, the patient’s hypertension and adrenal lesions were not investigated in the context of this hereditary syndrome for over a decade. This underscores a critical clinical failure: a known family history of a genetic disorder must aggressively prompt systematically screening, even for common symptoms like hypertension. Throughout this prolonged diagnostic delay, the patient underwent repeated evaluations for hypertension and incidentally detected masses, yet VHL syndrome remained undiagnosed until a catastrophic aortic dissection occurred. Critically, the patient conceived a daughter during this diagnostic gap, perpetuating genetic risk; (2) Another unpublished historical case illustrated that a male patient underwent sequential surgeries for retinal and cerebellar hemangioblastomas over 10 years preceding his eventual VHL diagnosis. Despite these pathognomonic lesions, the underlying syndrome was unrecognized, culminating in the birth of three biological children prior to genetic counseling (**Supplementary Figure 4**). Both cases highlight the consequences of delayed diagnosis—progeny inheriting a 50% risk of VHL mutations. Assisted with reproductive technologies (e.g., preimplantation genetic diagnosis) could mitigate this risk but require prior VHL syndrome recognition. There is a systematic review of VHL’s rare initial presentations and complications (**Supplementary Table 3**), which underscores the imperative for heightened clinical suspicion. Early recognition not only optimizes patient outcomes but also enables ethical reproductive planning through timely genetic counseling.

### Targeted therapy: a promising frontier in VHL syndrome management

As a paradigm of precision medicine, targeted therapy achieves disease modulation through molecular-level identification and inhibition of critical oncogenic drivers. VHL syndrome, caused by germline mutations in the VHL gene, leads to inactivation of the VHL protein’s tumor-suppressive function. This molecular defect results in pathological stabilization and accumulation of hypoxia-inducible factor-2α (HIF-2α), which orchestrates downstream oncogenic pathways including VEGF, PDGF, and EPO, thereby driving tumorigenesis in multiple organs (e.g., kidneys, pancreas, and CNS). Building on this mechanistic rationale, HIF-2α inhibitors and anti-angiogenic agents have emerged as a breakthrough therapeutic strategy. Multiple phase II/III trials have demonstrated their ability to suppress tumor progression in VHL patients (14, 15). In our clinical practice, a 30-year-old VHL patient with bilateral renal tumors and retroperitoneal metastases achieved radiologically confirmed tumor regression (**Supplementary Figure 5**) after anlotinib treatment, highlighting the clinical value of targeting this pathway. In the context of the case presented herein, targeted therapy remains a feasible option following surgical resolution of life-threatening emergencies.

## Conclusion

This case highlights three pivotal clinical insights for managing VHL syndrome with multisystem involvement: First, VHL-associated pathologies (e.g., pheochromocytoma-driven hypertension) may confer unique susceptibility to aortic dissection. Second, systematic screening for catecholamine-secreting tumors is imperative in patients with resistant hypertension to mitigate cardiovascular crises inherent to this multisystem disorder. Third, MDT care frameworks—integrating genetic, surgical, and oncologic expertise—are essential to resolve therapeutic dilemmas posed by competing life-threatening conditions. These findings reinforce that early recognition of multisystem complexity and proactive MDT coordination are critical to optimizing outcomes in VHL syndrome. Furthermore, this case reinforces the critical importance of screening for secondary hypertension, particularly in cases of early-onset, resistant, or paroxysmal hypertension, to identify underlying and potentially treatable conditions such as pheochromocytoma before catastrophic complications occur.

## Data Availability

The original contributions presented in the study are included in the article/**Supplementary Material**. Further inquiries can be directed to the corresponding authors.

## References

[1] Maher ER Neumann HP Richard S. von Hippel-Lindau disease: a clinical and scientific review. Eur J Hum Genet. (2011) 19:617–23. doi: 10.1038/ejhg.2010.175, PMID: 21386872 PMC3110036

[2] Nordstrom-O'Brien M van der Luijt RB van Rooijen E van den Ouweland AM Majoor-Krakauer DF Lolkema MP. Genetic analysis of von Hippel-Lindau disease. Hum Mutat. (2010) 31:521–37. doi: 10.1002/humu.21219, PMID: 20151405

[3] Binderup MLM Smerdel M Borgwadt L Beck Nielsen SSB Madsen MG Møller HU. von Hippel-Lindau disease: Updated guideline for diagnosis and surveillance. Eur J Med Genet. (2022) 65:104538. doi: 10.1016/j.ejmg.2022.104538, PMID: 35709961

[4] Kaelin WG Jr. Von Hippel-Lindau disease: insights into oxygen sensing, protein degradation, and cancer. J Clin Invest. (2022) 132:e162480. doi: 10.1172/JCI162480, PMID: 36106637 PMC9479583

[5] Poulsen ML Budtz-Jørgensen E Bisgaard ML. Surveillance in von Hippel-Lindau disease (vHL). Clin Genet. (2010) 77:49–59. doi: 10.1111/j.1399-0004.2009.01281.x, PMID: 19863552

[6] Hong B Ma K Zhou J Zhang J Wang J Liu S. Frequent mutations of VHL gene and the clinical phenotypes in the largest chinese cohort with von hippel-lindau disease. Front Genet. (2019) 10:867. doi: 10.3389/fgene.2019.00867, PMID: 31620170 PMC6759728

[7] Kalekar T Kumar SP Pachva A. Late-onset manifestations of von hippel-lindau syndrome: A case report. Cureus. (2024) 16:e62756. doi: 10.7759/cureus.62756, PMID: 39036180 PMC11260203

[8] Gong Y Taraif S Mazur I Aneja A Huang M Somers DL. Ovarian steroid cell tumor associated with von Hippel-Lindau syndrome: a report of two cases and literature review. Int J Clin Exp Pathol. (2022) 15:332–7., PMID: 36106071 PMC9441858

[9] Anderson S. Volatile hypertensive crisis secondary to pheochromocytoma: A case report of von hippel-lindau syndrome. J Pediatr Health Care. (2020) 34:264–72. doi: 10.1016/j.pedhc.2020.01.002, PMID: 32143938

[10] Harris KM Nienaber CA Peterson MD Woznicki EM Braverman AC Trimarchi S. Early mortality in type A acute aortic dissection: insights from the international registry of acute aortic dissection. JAMA Cardiol. (2022) 7:1009–15. doi: 10.1001/jamacardio.2022.2718, PMID: 36001309 PMC9403853

[11] House-Fancher MA. Aortic dissection: pathophysiology, diagnosis, and acute care management. AACN Clin Issues. (1995) 6:602–13; quiz 683-4. doi: 10.1097/00044067-199511000-00010, PMID: 7493263

[12] Pan J Jabarkheel R Huang Y Ho A Chang SD. Stereotactic radiosurgery for central nervous system hemangioblastoma: systematic review and meta-analysis. J Neurooncol. (2018) 137:11–22. doi: 10.1007/s11060-017-2697-0, PMID: 29204841

[13] Zare A Zare A Soltani Khaboushan A Hajikarimloo B Sheehan JP. Stereotactic radiosurgery in the management of central nervous system hemangioblastomas: a systematic review and meta-analysis. Neurosurg Rev. (2025) 48:303. doi: 10.1007/s10143-025-03454-9, PMID: 40091097 PMC11911270

[14] Jonasch E Donskov F Iliopoulos O Rathmell WK Narayan VK Maughan BL. Belzutifan for renal cell carcinoma in von hippel-lindau disease. N Engl J Med. (2021) 385:2036–46. doi: 10.1056/NEJMoa2103425, PMID: 34818478 PMC9275515

[15] Jonasch E McCutcheon IE Waguespack SG Wen S Davis DW Smith LA. Pilot trial of sunitinib therapy in patients with von Hippel-Lindau disease. Ann Oncol. (2011) 22:2661–6. doi: 10.1093/annonc/mdr011, PMID: 22105611 PMC4542805

