# Supplementary material for: Case report: Von Hippel-Lindau syndrome with multisystem involvement: a therapeutic dilemma
Source: Front Oncol. 2025 Oct 14;15:1633911. doi: 10.3389/fonc.2025.1633911 (PMC12558800; doi:10.3389/fonc.2025.1633911)
Supplement: Supplementary file 1 [file Table1.docx]

**Supplementary Table.1 Plasma catecholamine and metanephrine profile of the patient.**

| **Index** | **Measured value** | **Reference range** | **State** |
| --- | --- | --- | --- |
| DA (pmol/L) | 84.0 | ≤195.7 | normal |
| E (pmol/L) | 64.9 | ≤769.0 | normal |
| NE (pmol/L) | 3401.2 | 1182.8-10054.0 | normal |
| 3-MT (nmol/L) | ＜0.08 | ＜0.18 | normal |
| MN (nmol/L) | 0.09 | 0.08-0.51 | normal |
| **NMN** (nmol/L) | **3.25** | **0.12-1.18** | **elevate** |

Abbreviations: DA: Dopamine, E: Epinephrine, NE: Norepinephrine, 3-MT: 3-Methoxytyramine, MN: Metanephrine, NMN: Normetanephrine
