# Supplementary material for: Case report: Von Hippel-Lindau syndrome with multisystem involvement: a therapeutic dilemma
Source: Front Oncol. 2025 Oct 14;15:1633911. doi: 10.3389/fonc.2025.1633911 (PMC12558800; doi:10.3389/fonc.2025.1633911)
Supplement: Supplementary file 2 [file Table2.docx]

Supplementary Table 2. Published cases of von Hippel-Lindau (VHL) syndrome with diagnostic delays despite characteristic clinical and genetic manifestations.

| **Code** | **Age (years)** | **Sex** | **Delay (years)** | **Presenting Symptoms/VHL Features** | **Confirmed VHL Manifestations** | **Title** | **Journal** | **Year** | **PMID** |
| --- | --- | --- | --- | --- | --- | --- | --- | --- | --- |
| 1 | 41 | M | 15 | ccRCC, cerebral HBL, and the family history of VHL syndrome. | Pancreatic neuroendocrine tumor | A case report of cerebellar hemangioblastoma simulated brain metastasis shown by magnetic resonance imaging | Medicine (Baltimore) | 2024 | 38335432 |
| 2 | 46 | M | 16 | Parietal lobe HBL and the family history of VHL syndrome | Pancreatic neuroendocrine tumors and RCC | Surgical resection of double advanced pancreatic neuroendocrine tumors with multiple renal cell carcinoma associated with von Hippel- Lindau disease | Clin J Gastroenterol | 2024 | 38693425 |
| 3 | 64 | M | 3 | ccRCC | Intracranial lateral ventricular trigone HBL | Supratentorial Collision Tumor of Hemangioblastoma and Metastatic Clear Cell Renal Cell Carcinoma in a Patient with von Hippel-Lindau  Disease | Case Rep Oncol | 2023 | 37900808 |
| 4 | 39 | M | 23 | A history of benign brain tumors and the family history of VHL syndrome | Pancreatic cysts, renal cysts, gallbladder, cerebellar hypodense lesion, suspicious pheochromocytomas, and the family history of VHL syndrome. | Total pancreatoduodenectomy for multiple pancreatic cysts in von Hippel-Lindau disease presenting as obstructive jaundice: A case report | Int J Surg Case Rep | 2023 | 37454549 |
| 5 | 23 | F | 5 | Retinal hemangioma | Pancreatic neuroendocrine tumor with hepatic metastases | Co-occurrence of VHL and SDHA Pathogenic Variants: A Case Report | Front Oncol | 2022 | 35875079 |
| 6 | 19 | F | 5 | Fever and headache with high blood pressure | Pheochromocytoma, paraganglioma, pancreatic and lung neuroendocrine tumor. | Identification of a VHL gene mutation in atypical Von Hippel-Lindau syndrome: genotype–phenotype correlation and gene therapy perspective | Cancer Cell Int | 2021 | 34923986 |
| 7 | 38 | F | 20 | Blurred vision and dizziness | Renal cysts, mucus cyst, pancreatic polycyst with partial consolidation, islet cell tumors, polycysts in both kidneys, RCC, and left adrenal adenoma. | Effect of clarithromycin in von Hippel-Lindau syndrome: a case report | J Int Med Res | 2019 | 30178691 |
| 8 | 54 | M | 6 | Bilateral cerebellum HBL | RCC, polycystic kidneys, and liver cysts. | Novel gene mutation in von Hippel-Lindau disease - a report of two cases | BMC Med Genet | 2019 | 31823746 |
| 9 | 38 | M | 3 | Dizziness, headache and brain stem HBL | RCC and multifocal spinal HBL | Pedigree analysis, diagnosis and treatment in Von Hippel-Lindau syndrome: A report of three cases | Oncol Lett | 2018 | 29616089 |
| 10 | 40 | M | 11 | Severe hypertension and bilateral pheochromocytomas | RCC, cerebellum HBL, and a VHL mutation c.239G>T; p.(Ser80Ile). | A retrospective case study of sunitinib treatment in three patients with Von Hippel-Lindau disease | Cancer Biol Ther | 2018 | 29947576 |
| 11 | 35 | F | 14 | Cerebral HBL | Pancreatic solid tumors , pancreatic cystic lesions, RCC, and a mutation of exon 1 of VHL gene (233A>T). | Serous cystic neoplasms of the whole pancreas in a patient with von Hippel-Lindau disease | Intern Med | 2011 | 21673464 |
| 12 | 37 | F | 15 | Low back pain with paresthesias in her lower extremities and hands, as well as occipital  headaches for 9 months. | Spinal cord tumors, RCC, cystic kidneys bilaterally, pancreatic cysts and pancreatic solid nodule. | Should Endolymphatic Sac Tumors Be Considered Part of the Von Hippel-Lindau Complex? Pathology Case Report | Neurosurgery | 1997 | 9092862 |

Abbreviations: M: male, F: female, RCC: renal cell carcinoma, ccRCC: clear cell renal cell carcinoma, HBL: hemangioblastoma. Journal title abbreviations conform to the ISO 4 standards.
