# Supplementary material for: Case report: Von Hippel-Lindau syndrome with multisystem involvement: a therapeutic dilemma
Source: Front Oncol. 2025 Oct 14;15:1633911. doi: 10.3389/fonc.2025.1633911 (PMC12558800; doi:10.3389/fonc.2025.1633911)
Supplement: Supplementary file 3 [file Table3.docx]

Supplementary Table 3. Summary of published von Hippel-Lindau (VHL) syndrome cases with rare initial presentations and complications.

| **Code** | **Age**  **(years)** | **Sex** | **Typical VHL Manifestations** | **Initial Symptom** | **Rare Comorbidity** | **Diagnostic Methods** | **Treatment & Outcome** | **Genetic Mutation** | **Title** | **Journal** | **Year** | **PMID** |
| --- | --- | --- | --- | --- | --- | --- | --- | --- | --- | --- | --- | --- |
| 1 | 19 | F | HBL, renal and pancreatic cysts. | Typical central obesity, wide purple striae on the abdomen, fatigue, hair loss, dryness of mouth, and polydipsia. | ACTH-secreting adenomas | CT, MRI, histopathologic analysis, laboratory tests, and genetic analysis. | Surgical resection | The first exon of the VHL gene (c.227_229del, p.76delF) | A Corticotropin-Secreting Adenoma in the Setting of von Hippel- Lindau Disease | JCEM Case Rep | 2025 | 40213016 |
| 2 | 31 | F | Lerebellar vermian HBL | Hearing loss, inability to close left eyelid and left hemifacial spasm. | Left cerebellopontine angle ELST with low-grade adenocarcinoma | CT, MRI, and histopathologic analysis | Surgical resection | NR | Management of Pregnancy in a Case of Endolymphatic Sac Tumour as a Part of Von Hippel-Lindau Syndrome Complex: A Case Report and Review of Literature | J Obstet Gynaecol India | 2025 | 40092392 |
| 3 | 25 | F | Multiple renal, hepatic, and pancreatic lesions. | Left arm weakness and numbness | Multiple HBL in intramedullary and intra-axial | MRI, genetic testing, and histopathologic examination. | Surgical resection and drug therapy | A heterozygous deletion of exons 2-3 in the VHL gene | Exceptional and Sustained Response to Belzutifan in Von Hippel- Lindau Disease-Associated Central Nervous System Hemangioblastoma | Cureus | 2024 | PMID: 38406059 |
| 4 | 23 | F | Pancreatic cysts, cerebellum and cervical spinal hemangioblastomas. | NR | Type 1 gastric neuroendocrine tumor | MRI, upper gastrointestinal endoscopy, PET- CT, histopathologic analysis, genetic analysis, and laboratory tests. | Surgical resection | c.484 T > C/p.(Cys162Arg) mutation of the VHL  gene | When rare diseases crisscross within the same patient: von Hippel-Lindau and type 1 gastric neuroendocrine tumor | Hormones (Athens) | 2024 | 38619811 |
| 5 | 19 | F | Cerebellar HBL, cervical spinal HBL, and brainstem HBL. | Major depression | NR | MRI and genetic analysis | Surgical resection | A mutation [p.Asn78Ser (c.233A>G)  HETEROZYGOT] | A Von Hippel-Lindau Syndrome Case Diagnosed While Hospitalized for Major Depression | Psychiatry Clin Psychopharmacol | 2024 | 39165895 |
| 6 | 42 | F | Pheochromocytoma, hypertension, cerebellar, optic nerve, retina, and medular HBL. | Headaches radiating to cervical region, burning sensation, localized numbness, dizziness,vomiting, progressive worsening, and gait disorder. | Medular HBL | CT and MRI | Symptomatic treatment of hydrocephalus and optional radiotherapy | NR | Optic nerve haemangioblastoma in association with von Hippel- Lindau syndrome: case report and literature review | BJR Case Rep | 2024 | 38486681 |
| 7 | 23 | F | HBL in retinal, brain and spinal cord. | Visual deterioration | Pancreatic HBL | Fundus photography, CT, and MRI. | Drug thearapy | NR | Belzutifan-induced regression of retinal capillary hemangioblastoma: A case-series | Am J Ophthalmol Case Rep | 2024 | 38374949 |
| 8 | 39 | M | Pancreatic and renal cysts, benign brain tumors, cerebellum ametabolic hypodense lesion, suspicious pheochromocytomas, and the family history of VHL syndrome. | Jaundice and pruritus | Obstructive jaundice | MRI, MRCP, CT, and histopathologic analysis. | Surgical resection | NR | Total pancreatoduodenectomy for multiple pancreatic cysts in von Hippel-Lindau disease presenting as obstructive jaundice: A case report | Int J Surg Case Rep | 2023 | 37454549 |
| 9 | 30 | M | Retinal HBL and pancreatic tumours | NR | Idiopathic interstitial pneumonia | CT, echocardiogram, electromyography, histopathologic analysis, and genetic analysis. | Surgical resection, drug therapy, and waiting for transplantation. | VHL P86A mutations of exon 1 | Idiopathic interstitial pneumonia in a patient with von Hippel– Lindau syndrome: a first case | ERJ Open Res | 2023 | 38020566 |
| 10 | 56 | M | Cerebellar HBL, RCC, pancreatic cystic lesions, and adrenal gland cystic lesions. | Severe abdominal pain without any trigger | Bilateral epididymides solid lesions | CT, MRI, PET/CT, and histopathologic  analysis. | NR | NR | 18 F-PSMA-1007 PET/CT in a Case of Von Hippel-Lindau  Syndrome | Clin Nucl Med | 2023 | 37844561 |
| 11 | 15 | M | Pheochromocytoma and hypertension | A six-month history of severe headaches and vomiting | Bilateral pheochromocytoma with a splenic lesion and left-sided subdiaphragmatic paraganglioma | CT, PET/CT, laboratory tests, and  histopathologic analysis. | Surgical resection | NR | Bilateral Pheochromocytoma and Paraganglioma Tumors Due to Von Hippel-Lindau Syndrome in a 15-Year-Old Boy: A Case Report | Cureus | 2023 | 38021838 |
| 12 | 19 | F | Cerebellar HBL | Menstrual irregularities | Ovarian steroid cell tumor | MRI, US, and histopathological analysis. | Surgical resection | NR | Ovarian steroid cell tumor associated with von Hippel-Lindau syndrome: a report of two cases and literature review | Int J Clin Exp Pathol | 2022 | 36106071 |
| 13 | 39 | F | Retinal and cerebellar HBL, renal cysts and ccRCC. | Abnormal uterine bleeding and elevated testosterone | Ovarian steroid cell tumor | US, CT, and histopathological analysis. | Surgical resection | NR | Ovarian steroid cell tumor associated with von Hippel-Lindau syndrome: a report of two cases and literature review | Int J Clin Exp Pathol | 2022 | 36106071 |
| 14 | 35 | M | Multiple benign CNS and visceral tumors | NR | Sinonasal renal cell-like adenocarcinoma | CT, MRI, and nasal endoscopic biopsy. | Surgical resection, radiotherapy, and no recurrence one year from diagnosis. | A mutation in exon 3 of VHL, R161P (c.482G >  C) | Sinonasal renal cell-like adenocarcinoma arising in von Hippel Lindau (VHL) syndrome | Oral Oncol | 2022 | 34998175 |
| 15 | 35 | M | Cerebellar HBL, hypertension, pheochromocytoma, kidney and pancreatic cysts. | Dyspnea | Dilated cardiomyopathy | ECG examinations, MRI, and CT. | Surgical resection and drug therapy | A missense mutation of the VHL gene, c.269  A > T (p.Asn90Ile) | Von Hippel-Lindau syndrome with a rare complication of dilated cardiomyopathy: a case report | BMC Cardiovasc Disord | 2022 | 36401171 |
| 16 | 36 | M | Retinal HBL, RCC, pancreatic cysts, and suspicious pheochromocytoma. | Painless diminution of vision | Viral conjunctivitis | Funduscopy, MRI, and PET/CT. | Drug therapy | NR | Incidental Detection of Viral Conjunctivitis on 68Ga-DOTANOC PET/CT in a Patient Suspected With Von Hippel–Lindau Syndrome | Clin Nucl Med | 2022 | 35195580 |
| 17 | 20 | M | Retinal HBL, liver tumor, and the family history of VHL syndrome. | A half-year vision loss with the left eye | Polycythemia and central retinal vein occlusion | Fundus fluorescein angiography, MRI, and genetic analysis. | Surgical resection | VHL c.208G > A | Von Hipple-Lindau disease complicated with central retinal vein occlusion: a case report | BMC Ophthalmol | 2022 | 36384467 |
| 18 | 45 | M | RCC, cerebellar HBL, and the family history of VHL syndrome. | NR | Gastric neuroendocrine tumors | CT, laboratory tests, endoscopic findings, histopathologic analysis, and genetic analysis. | Surgical resection | VHL gene exon1, S65W (c.194 C>G) | The First Case of Gastric Neuroendocrine Tumors Induced by a Proton Pump Inhibitor in von Hippel-Lindau Disease | Intern Med | 2022 | 35135919 |
| 19 | 20 | NR | Retinal capillary hemangioblastoma | Headache and blurred vision | Suprasellar paraganglioma | MRI, digital subtraction angiography, fundus fluorescein angiography, and histopathologic analysis. | Surgical resection and radiotherapy | NR | Suprasellar paraganglioma in a clinical setting of von Hippel- Lindau syndrome | BMJ Case Rep | 2022 | 35321910 |
| 20 | 25 | F | Cerebellar HBL, pancreatic neuroendocrine tumor, pancreatic cysts, RCC, and the family history of VHL syndrome. | Lower back pain | Retroperitoneal paraganglioma at the bifurcation of the common iliac artery | CT, MIBG scintigraphy, laboratory tests, histopathologic analysis, and genetic analysis. | Surgical resection | A heterozygous mutation of VHL (NM_000551.4), p.Ser65Trp by single nucleotide substitution of c.194C>G in exon 1 | Retroperitoneal paraganglioma with loss of heterozygosity of the von Hippel-Lindau gene: a case report and review of the literature | Endocr J | 2022 | 35466127 |
| 21 | 17 | M | Retinal and cerebellar HBL, solid cystic cerebellar mass, the endolymphatic sac, and left cerebellopontine angle tumor. | Hearing loss associated with visual loss and worsening headache | Left cerebellopontine angle tumor, the endolymphatic sac and cerebellar hemangioblastoma. | MRI, CT, retinal HBL, histopathologic analysis, and genetic analysis. | Surgical resection | NR | Synchronous Presentation of Rare Brain Tumors in Von Hippel- Lindau Syndrome | Diagnostics (Basel) | 2021 | 34072835 |
| 22 | 22 | M | Pheochromocytoma, kidney cysts, suspcious RCC, pancreatic cystadenomas, epididymis lesion, thyroid cystic nodules, and brainstem HBL. | Infertility | Infertility | CT, US, and MRI. | Surgical resection | NR | Anesthetic management of bilateral pheochromocytoma resection in Von Hippel-Lindau syndrome: A case report | World J Clin Cases | 2021 | 34046474 |
| 23 | 19 | F | Pheochromocytoma, paraganglioma, and pancreatic neuroendocrine tumor. | Fever and headache with high blood pressure | Lung neuroendocrine tumor | CT, histopathologic analysis, and genetic analysis. | Surgical resection and drug therapy | c.500G > A， p.Arg167Gln | Identification of a VHL gene mutation in atypical Von Hippel- Lindau syndrome: genotype–phenotype correlation and gene therapy perspective | Cancer Cell Int | 2021 | 34923986 |
| 24 | 19 | F | HBL in the fourth ventricle causing hypertensive hydrocephalus and pancreatic cystic lesions | Severe weight loss and anorexia | NR | CT，MRI，X-ray， Esophagogastroduodenography and upper digestive endoscopy, and genetic analysis. | VP shunt with radiotherapy (tumor controlled), persistent dysphagia, and sporadic nausea. | A nonsense VHL mutation (single-nucleotide change causing a premature stop codon in exon 3: c.481C>T; p.Arg161*) | Anorexia as the first clinical manifestation of von Hippel-Lindau syndrome | Mol Clin Oncol | 2020 | 32974018 |
| 25 | 32 | M | Medulla hemangioblastoma, RCC, and pheochromocytoma. | Right ankle pain | Clear cell chondrosarcoma of the talus | CT, bone scan, and histopathologic analysis. | Surgical resection and radiotherapy | NR | Clear cell chondrosarcoma of the talus in Von Hippel-Lindau disease: a rare tumor in an unusual location and uncommon co- presentation | Int J Clin Exp Pathol | 2020 | 32211108 |
| 26 | 11 | F | Hypertension, plasma/urine catecholamines (elevated), and pheochromocytoma. | Hypertensive emergency with ocular/cardiac sequelae | Ovarian steroid cell tumor | CT, MRI, laboratory tests, histopathologic analysis, and genetic analysis | Surgical resection | A c.695G>A (p.Arg161Gln) pathogenic  missense mutation in exon 3 of the VHL gene | Ovarian Steroid Cell Tumor in an Adolescent With Von Hippel- Lindau Syndrome: A Case Report and Review of the Literature | Int J Gynecol Pathol | 2020 | 31433374 |
| 27 | 22 | F | Brainstem and juxtapapillary retinal capillary HBL | Juxtapapillary retinal capillary HBL and old hemiretinal vein occlusion. | Childhood glaucoma | Color fundus photograph, spectral domain optical coherence tomography, fundus fluorescein angiography, and MRI. | A deep coma and passed away after the surgical resection | NR | Retinal capillary hemangioblastoma and hemiretinal vein occlusion in a patient with primary congenital glaucoma: A case report | Saudi J Ophthalmol | 2019 | 31920453 |
| 28 | 10 | F | Pheochromocytoma and pancreatic neuroendocrine tumor | Intermittent fever | Pancreatic neuroendocrine tumor | Abdominal MRI, laboratory tests, PET/CT, and  genetic analysis. | Surgical resection | A heterozygous missense mutation c. 386 T>G  (p.Leu129Pro) | Two Childhood Pheochromocytoma Cases due to von Hippel- Lindau Disease, One Associated with Pancreatic Neuroendocrine Tumor: A Very Rare Manifestation | J Clin Res Pediatr Endocrinol | 2018 | 29022557 |
| 29 | 38 | M | Brainstem HBL and ccRCC | Dizziness and headache | Multifocal spinal HBL | CT, MRI, histopathologic analysis, and genetic analysis. | Surgical resection | A mutation of the VHL gene, c.227_229delTCT (p.Phe76del) | Pedigree analysis, diagnosis and treatment in Von Hippel-Lindau syndrome: A report of three cases | Oncol Lett | 2018 | 29616089 |
| 30 | 12 | M | Pheochromocytoma and elevated plasma metanephrines. | Asymptomatic | Right thoracic paravertebral paraganglioma and lung ectopic pheochromocytomas | CT and laboratory examination | Surgical resection | NR | Metastatic Pheochromocytoma in an Asymptomatic 12-Year-Old With von Hippel-Lindau Disease | Urology | 2018 | 9545044 |
| 31 | 42 | F | Brain and spinal cord HBL, pheochromocytoma, pancreatic neuroendocrine tumors, and the family history of VHL syndrome | A right-sided neck mass | Low-grade mucoepidermoid carcinoma | Histopathologic analysis and genetic analysis | Surgical resection | An exon 3 mutation of VHL gene (Nt713G>A) | Case of parotid mucoepidermoid carcinoma: Expanding the spectrum of von Hippel-Lindau–related neoplasms | Head Neck | 2017 | 28006088 |
| 32 | 30 | F | Cervical spine HBL, pancreatic cysts, liver cysts, renal cysts, and the family history of VHL syndrome. | Gradual onset weakness in all four limbs | RCC with isolated metastasis to sigmoid mesentery | US, CT, MRI, and histopathologic analysis. | Surgical resection | NR | Renal cell carcinoma with isolated metastasis to sigmoid mesentery: a rare resectable combination | BMJ Case Rep | 2017 | 28918405 |
| 33 | 29 | F | Cerebral HBL, RCC, and pancreatic cysts. | NR | Pancreatic gastrinoma | MRI, PET/CT, and histopathologic analysis. | NR | NR | Management of pancreatic gastrinoma associated with Von Hippel-Lindau disease: a case report | Rev Esp Enferm Dig | 2017 | 27055912 |
| 34 | 54 | F | Pancreatic cysts, renal cyst, spinal HGB, RCC, and hypertension. | Acute altered mental status and respiratory distress requiring intubation | Breast cysts | MRI, CT, and cerebral angiogram. | Radiotherapy | NR | Recurrent hemorrhage in hemangioblastoma involving the posterior fossa: Case report | Surg Neurol Int | 2017 | 28713626 |
| 35 | 43 | M | RCC | Primary infertility and non-obstructive azoospermia | Epididymal papillary cystadenocarcinoma metastasising to the testis | US, CT, and histopathologic analysis. | Surgical resection | NR | Epididymal papillary cystadenocarcinoma metastasising to the testis in a patient with infertility managed with Onco-microTeSE | J Surg Case Rep | 2016 | 27887012 |
| 36 | 74 | M | Hypertension, RCC, and HBL. | Ait disturbance and tingling sensation of both legs | Colorectal adenocarcinoma | CT, MRI, and histologic examination | Surgical resection and radiotherapy | VHL gene showed p.Glu70Lys (c.208G > A)  mutation in exon 1 | A Case of von Hippel-Lindau Disease with Colorectal Adenocarcinoma, Renal Cell Carcinoma and Hemangioblastomas | Cancer Res Treat | 2016 | 25715769 |
| 37 | 38 | M | Pheochromocytomas and paraganglioma | Abdominal pain in the epigastric region and generalized itching | Pancreatic mixed serous neuroendocrine neoplasm with clear cells | US, MRI, CT, and histopathologic analysis. | Surgical resection and died after resection for postoperative adrenal crisis (1month) | NR | Pancreatic mixed serous neuroendocrine neoplasm with clear cells leading to diagnosis of von Hippel Lindau disease | Pathol Res Pract | 2016 | 27161305 |
| 38 | 25 | M | Retinal capillary HBL and pancreatic cysts | Diffuse muscular pain, muscle weakness, and brown-colored urine. | CPT2 Deficiency | CT, histopathologic analysis, and genetic analysis. | Drug therapy | Chromosome 3p25.3; c. 256 C>G; p.P86A  mutation | Coexistence of VHL Disease and CPT2 Deficiency: A Case Report | Cancer Res Treat | 2016 | 27034144 |
| 39 | 17 | F | Cerebellar HBL and pheochromocytoma | Pain in her right eye | Terson’s Syndrome | Fundus photography and fluorescein angiography | Surgical resection and drug therapy | NR | Terson's Syndrome in a Patient with von Hippel-Lindau Disease | Optom Vis Sci | 2016 | 27309526 |
| 40 | 61 | F | Pancreatic neuroendocrine tumor, pheochromocytomas, spinal HBL, and renal cystic lesions. | NR | Pulmonary mucosa-associated lymphoma | CT, PET/CT, histopathologic analysis, and  genetic analysis. | Surgical resection | Exon III mutation | Pulmonary mucosa-associated lymphoma in a patient with von Hippel-Lindau disease | J Surg Case Rep | 2015 | 26173437 |
| 41 | 30 | M | Retinal HBL and pheochromocytomas | NR | Pulmonary carcinoid with cranial metastasis | MRI, CT, histopathologic analysis, and genetic analysis. | Surgical resection | A mutation of the VHL gene (Tyr98His, cDNA  292T>C) | Von hippel-lindau disease associated pulmonary carcinoid with cranial metastasis | J Clin Endocrinol Metab | 2014 | 24878057 |
| 42 | 64 | F | Cerebellum HBL, RCC, pancreatic cysts, and the family history of VHL syndrome. | NR | Lung adenocarcinomas | CT, histopathologic analysis, and genetic analysis. | Surgical resection | C to T substitution in exon 1 that changes the identity of codon 65 from serine (TCG) to leucine (TTG) | Multiple lung adenocarcinomas associated with von hippel-lindau disease | Ann Thorac Surg | 2014 | 25282218 |
| 43 | 18 | M | Pheochromocytoma, para-aortic paraganglioma, and endocrine tumor. | Physical examination | A superior mediastinal neurogenic tumor | X-ray, CT, MRI, histopathologic analysis, and genetic analysis. | Surgical resection | A mutation of the VHL gene (exon2 TTT → TGT) | Superior mediastinal paraganglioma associated with von Hippel- Lindau syndrome: report of a case | World J Surg Oncol | 2014 | 24678933 |
| 44 | 42 | F | HBL | Intermittent headaches and dizziness, without nausea and vomiting. | Co-existing ELST and HBL in the posterior cranial fossa | CT, MRI, and genetic analysis. | Surgical resection | 291st site of Exon 1 was insertion of 10 nucleotides (GCCGCAGCCC) | Endolymphatic sac tumor with von Hippel-Lindau disease: report of a case with atypical pathology of endolymphatic sac tumor | Int J Clin Exp Pathol | 2014 | 24966975 |
| 45 | 18 | M | Pheochromocytoma and pancreatic endocrine tumor | NR | Para-aortic paraganglioma | CT, X-ray, MRI, histopathologic analysis, and genetic analysis. | Surgical resection | A mutation of the VHL gene (exon2 TTT → TGT) | Superior mediastinal paraganglioma associated with von Hippel- Lindau syndrome: report of a case | World J Surg Oncol | 2014 | 24678933 |
| 46 | 64 | M | Pheochromocytoma, hepatic cysts, and RCC. | Intermittent abdominal pain, hypertension, loss of appetite and constipation | Colon adenocarcinoma | CT, colonoscopy, histologic examination, US, fundo  scopy, and PET/CT. | Surgical resection | A mutation in exon 1 at the level of codon 25  (CCT>CTT; Pro>Leu in heterozygosis) | von Hippel Lindau disease with colon adenocarcinoma, renal cell carcinoma and adrenal pheochromocytoma | Intern Med | 2013 | 23857093 |
| 47 | 56 | M | Cerebellar HBL | Sudden onset of lumbago, neck pain, headache, and transient weakness in both lower extremities. | Intra-extradural dumbbell-shaped HBL  subarachnoid hemorrhage in the cauda equina | MRI and CT | Surgical resection | NR | Intra-extradural dumbbell-shaped hemangioblastoma manifesting  as subarachnoid hemorrhage in the cauda equina | Neurol Med Chir (Tokyo) | 2012 | 23006882 |
| 48 | 36 | M | Cerebellar HBL and renal cysts | Dull aching pain and heaviness in the right flank, raised blood pressure with fine tremors of both hands. | Phaeochromocytoma | Laboratory examination, US, CT, and histopathologic analysis. | Surgical resection | NR | Adrenal haemangioblastoma presenting as phaeochromocytoma: a rare manifestation of extraneural hemangioblastoma | Endocr Pathol | 2012 | 22544391 |
| 49 | 18 | M | Cerebellar HBL and the family history of VHL syndrome | Decreased visual acuity of the right eye | Pituitary adenoma | MRI, histopathologic analysis, laboratory tests, and genetic analysis. | Surgical resection and radiotherapy | The first exon of the VHL gene (c.340G > C,  p.Gly114Arg) | Von Hippel-Lindau disease and aggressive GH-PRL pituitary adenoma in a young boy | Ann Endocrinol (Paris) | 2012 | 22265326 |
| 50 | 49 | F | Retinal angiomatosis, renal cysts, and spinal cord HBL. | Pains in the second and third finger of the right hand | Carotid body paraganglioma | MRI, CT, and ophthalmological examination. | Surgical resection | The 393C>A (N131K) germline mutation in the  VHL gene | A case of carotid body paraganglioma and haemangioblastoma of the spinal cord in a patient with the N131K missense mutation in the VHL gene | Neurol Sci | 2011 | 21384277 |
| 51 | 23 | F | Spinal cord capillary HBL | Lower abdominal pain of unclear etiology | Bilateral papillary cystadenomata of the broad ligament | US, MRI, histopathologic analysis, and genetic analysis. | Surgical resection | A point mutation P86L on the exon 1 of the VHL gene | Bilateral papillary cystadenoma of the mesosalpinx: a rare manifestation of Von Hippel-Lindau disease | Arch Gynecol Obstet | 2010 | 20157715 |
| 52 | 28 | F | Pancreatic cysts, pancreatic neuroendocrine tumors, and ccRCC. | Virilization signs (clitoromegaly + hirsutism) and elevated T/FT/17-  OHP | Lipid Cell Tumors | US, CT, MRI, laboratory tests, and histopathologic analysis. | Surgical resection | NR | Lipid cell tumors in two women with von Hippel-Lindau syndrome | Obstet Gynecol | 2010 | 20664446 |
| 53 | 30 | F | Hypertension and pheochromocytoma | Uncontrolled hypertension | Thoracic paraganglioma | CT, histopathologic analysis, MIBG scintigraphy, laboratory tests, and genetic analysis. | Surgical resection | A heterozygous missense mutation c.482G>A in  exon 3 of the VHL gene | Concurrent bilateral pheochromocytoma and thoracic paraganglioma during pregnancy | Endocrine | 2010 | 20960261 |
| 54 | 54 | F | Cerebellar and spinal HBL, pancreatic cysts, and pancreatic hypervascular tumors. | NR | Serous cystic neoplasm in an intrapancreatic accessory spleen | CT and histopathologic analysis | Surgical resection | NR | Serous cystic neoplasm in an intrapancreatic accessory spleen | Pathol Int | 2010 | 20846266 |
| 55 | 26 | F | Cervical spine HBL and RCC | Polycystic ovarian syndrome | Lipid Cell Tumors | US, CT, laboratory tests, and histopathologic analysis. | Surgical resection | NR | Lipid cell tumors in two women with von Hippel-Lindau syndrome | Obstet Gynecol | 2010 | 20664446 |
| 56 | 53 | M | Renal cysts, RCC, pheochromocytomas, HBL in retinal, brain and spinal cord. | Neck pain and visual disturbances | Aggressive leptomeningeal hemangioblastomatosis | MRI and CT | Surgical resection and radiotherapy | NR | Aggressive leptomeningeal hemangioblastomatosis of the central nervous system in a patient with von Hippel-Lindau disease | AJNR Am J  Neuroradiol | 2009 | 19039053 |
| 57 | 4 | M | Optic nerve angioma (juxtapapillary capillary hemangioma) | Blurry vision | Optic nerve angioma (juxtapapillary capillary hemangioma) | Ophthalmic examination | Surgical resection | NR | Complex juxtapapillary capillary hemangioma: a case report | Optometry | 2008 | 18722963 |
| 58 | 31 | F | Pheochromocytoma | Biliary colic | Common bile duct carcinoid tumor | ERCP and pathological analysis | Surgical resection | NR | Carcinoid tumor of the common bile duct: a rare complication of von Hippel-Lindau syndrome | World J Gastroenterol | 2008 | 18300362 |
| 59 | 4 | M | Retinal HBL and endolymphatic sac tumor | Right hearing loss and seventh nerve palsy | Endolymphatic sac tumor | MRI, histopathologic analysis and genetic analysis. | Surgical resection | A VHL mutation in gene 3p25-26 | Endolymphatic sac tumor as initial manifestation of von hippel- lindau syndrome | Retin Cases Brief Rep | 2008 | 25390606 |
| 60 | 16 | F | Cerebellar and brainstem HBL | Abnormal early satiety resulting in growth and developmental arrest | Abnormal early satiety resulting in growth and developmental arrest | CT and MRI | Surgical resection, resulted in restoration of appetite, with rapid weight gain, growth in stature, and onset of menses. | NR | Pathological satiety caused by brainstem hemangioblastoma | J Neurosurg Pediatr | 2008 | 19035684 |
| 61 | 65 | M | RCC, multiple cysts in liver, spleen and pancreas. | Gross, painless hematuria, and clots. | Bilateral mutlicentric RCC with synchronous solitary urinary bladder metastasis | CT, histopathologic analysis, and genetic analysis. | Surgical resection | VHL chromosome 3 mutation | An unusual case of Von Hipple Lindau (VHL) syndrome with bilateral multicentric renal cell carcinoma with synchronous solitary urinary bladder metastasis | Int Urol Nephrol | 2007 | 17268895 |
| 62 | 20 | F | Cerebellar HBL and multiple cysts along the body | Nausea, weakness, vomiting, dizziness, and visual disturbance. | Pancreatic cystadenoma (giant) and bilateral ovarian cysts | Fundoscopy, CT, and histopathologic examination. | Surgical resection | NR | Pancreatic lesions in von Hippel-Lindau syndrome: report of a case | Surg Today | 2004 | 15221562 |
| 63 | 57 | M | Cerebellar HBL | 4-week history of nausea and dizziness | Primary hyperparathyroidism | MRI, CT, cerebral angiogram, histopathologic analysis, and laboratory examination. | Surgical resection | NR | Cerebellar hemangioblastoma associated with primary hyperparathyroidism--case report | Neurol Med Chir (Tokyo) | 2003 | 12627888 |
| 64 | 78 | F | Pheochromocytoma, RCC, and Spinal HBL. | Unstable blood pressure, headache and chest oppression | Primary Hyperparathyroidism | CT, MRI, laboratory examination, MIBG scintigraphg, histopathologic analysis, and genetic analysis. | Surgical resection | A variant in exon 1 that was caused by a transition A506->G in codon 98, resulting in Tyr293>Cys. | A case of von Hippel-Lindau disease with bilateral pheochromocytoma, renal cell carcinoma, pelvic tumor, spinal hemangioblastoma and primary hyperparathyroidism | Endocr J | 2002 | 12081237 |
| 65 | 20 | F | Cervical spinal cord HBL and the family history of VHL syndrome | Decrease in visual acuity | Persistent hyperplastic primary vitreous | Ophthalmoscopic examination and MRI | NR | NR | Persistent hyperplastic primary vitreous and von Hippel-Lindau disease: contribution of color Doppler ultrasonography | J Ultrasound Med | 2002 | 12008820 |
| 66 | 9 | M | Pheochromocytoma and hypertension | Post-traumatic convulsions | Multifocal paraganglioma partially involving the right suprarenal gland, abdominal paraganglia, and the left para-aortal retropleural region. | MIBG scintigraphy, MRI, X-ray, US, blood and urine analysis, and genetic analysis. | Surgical resection | A new point mutation on exon 1 (406 T→G) | Pheochromocytoma and multifocal functioning paraganglioma in a 9-year-old boy with von Hippel-Lindau disease | Urol Int | 2002 | 12053037 |
| 67 | 38 | M | Cerebellar HBL and RCC | Right upper quadrant pain | Clear cell carcinoid tumor of the gallbladder | CT and US | Surgical resection and renal transplantation | NR | Clear cell carcinoid tumor of the gallbladder: another distinctive manifestation of von Hippel-Lindau disease | Am J Surg Pathol | 2001 | 11688471 |
| 68 | 68 | F | Cerebellar hemangioma, fossa posterior hemangioma, ccRCC, multiorgan cysts  (renal/hepatic/splenic/pancreatic), and the family history of VHL syndrome. | High fever, pulmonary interstitial thick- ening and multiple hepatic lesions. | Hodgkin's disease | MRI, CT, histopathologic analysis, laboratory tests, X-ray, and bronchoscopy. | Surgical resection and chemotherapy | NR | Hodgkin's disease in a patient with Von Hippel-Lindau disease. A case report | Acta Clin Belg | 2000 | 11109642 |
| 69 | 40 | F | Phaeochromocytoma, renal cysts, brain stem and spinal cord enhancing masses. | A progressive history of neck and right arm pain associated with tingling and numbness in the right thumb and the sole of the right foot. | Cervical sensory nerve root HBL | MRI, histopathologic analysis, and genetic analysis. | Surgical resection | NR | Haemangioblastoma of a cervical sensory nerve root in Von Hippel-Lindau syndrome | Eur Spine J | 2000 | 11057539 |
| 70 | 22 | F | Retinal angiomatosis, cerebellar HBL, RCC, pancreatic cysts. | Lost her vision | RCC with metastasis to pancreatic tail | CT, US, celiac arteriogram, and histopathologic analysis. | Surgical resection | NR | Pancreatic lesions in von Hippel-Lindau syndrome: the coexistence of metastatic tumors from renal cell carcinoma and multiple cysts | Surg Today | 2000 | 10795874 |
| 71 | 21 | F | Renal cysts, HBL in retinal, brain and spinal cord. | Blurred vision | Postpartum cerebellar herniation | Fundus examination and MRI | Surgical resection | NR | Postpartum cerebellar herniation in von Hippel-Lindau syndrome | Am J Ophthalmol | 1999 | 10511048 |
| 72 | 22 | F | Retinal hemangioma, renal cysts, multiple pancreatic and renal nodules, and the family history of VHL syndrome | Loss of movement of the right upper limb and hemifacial syndrome of the right Parkinson 's syndrome | Parkinsonism hemisyndrome | CT, MRI, cerebral angiogram, and histopathologic analysis. | Surgical resection | NR | Parkinsonian hemi-syndrome as the initial manifestation of supratentorial cystic hemangioblastoma in a patient with Von Hippel-Lindau disease | Rev Neurol | 1998 | 9563091 |
| 73 | 37 | F | Spinal cord numerous tumors, ccRCC, large cystic kidneys, pancreatic cyst and pancreatic solid nodule. | Low back pain with paresthesias in lower extremities and hands, as well as occipital headaches. | ELST tumor | CT, MRI, histopathologic analysis, and SSCP analysis. | Surgical resection | NR | Should Endolymphatic Sac Tumors Be Considered Part of the Von Hippel-Lindau Complex? Pathology Case Report | Neurosurgery | 1997 | 9092862 |

| 74 | 32 | F | Spinal-medullary junction HBL, RCC, Pancreatic and renal cystic lesions. | NR | Pancreatic metastases from RCC | CT and histopathologic analysis | Surgical resection | NR | Pancreatic metastases from renal cell carcinoma in von Hippel-  Lindau disease | Clin Imaging | 1997 | 9117930 |
| --- | --- | --- | --- | --- | --- | --- | --- | --- | --- | --- | --- | --- |
| 75 | 46 | M | Cerebellar HBL and pheochromocytoma | A transient tinnitus | Normotensive bilateral pheochromocytoma | MRI, CT, and histopathologic analysis. | Surgical resection | NR | Normotensive bilateral pheochromocytoma with Lindau disease: case report | Endocr J | 1996 | 9075613 |
| 76 | 32 | M | Cerebellar HBL, pancreatic and renal cysts. | Nausea, vomiting, and constant epigastric pain. | Acute Pancreatitis and Evan's Syndrome | CT and US | ERCP and symptomatic treatment | NR | Von Hippel-Lindau disease complicated by acute pancreatitis and Evan's syndrome | Int J Pancreatol | 1995 | 8708400 |
| 77 | 24 | M | Cerebellar hemangioblastoma | Infertility | Bilateral clear cell papillary cystadenoma of the epididymides and infertility | CT and histopathologic analysis | Surgical resection | NR | Bilateral clear cell papillary cystadenoma of the epididymides presenting as infertility: an early manifestation of von Hippel- Lindau's syndrome | J Urol | 1985 | 3999214 |
| 78 | 29 | M | Cerebellar HBL and angiomatous lesions of the left eye | Infertility | Papillary cystadenoma of the epididymis | CT and histopathologic analysis | NR | NR | Bilateral papillary cystadenoma of the epididymis as a component of von Hippel-Lindau's syndrome: report of a case presenting as infertility | J Urol | 1985 | 3968752 |
| 79 | 56 | F | Retinal hemangioblastomas, pheochromocytoma, RCC, and multiple organ cysts(kidney, pancrea). | NR | Cerebellar astrocytoma and metastatic cakitonin- secreting islet cell carcinoma | CT, histopathologic analysis, and laboratory tests. | Surgical resection | NR | Metastatic islet cell tumor in von Hippel-Lindau disease | Am J Med | 1984 | 6331159 |
| 80 | 46 | M | Renal vascular tumors, renal cysts, frontal lobe vascular tumor, RCC, leptomeningeal HBL, and the family history of VHL syndrome. | Hematuria and a grand mal seizure | Leptomeningeal hemangioblastorna | Bilateral carotid arteriograms, bilateral selective renal arteriograms, and CT. | Surgical resection | NR | Supratentorial leptomeningeal hemangioblastoma | Neurology | 1978 | 566874 |

Abbreviations: M: male, F: female, NR: none report, RCC: renal cell carcinoma, ccRCC: clear cell renal cell carcinoma, HBL: hemangioblastoma, ACTH: adrenocorticotropic hormone, ELST: endolymphatic sac tumor, CT: computed tomography, MRI: Magnetic Resonance Imaging, US: ultrasound, SSCP: Single-strand conformation polymorphism, ECG: Electrocardiogram, MIBG: metaiodobenzylguanidine, PET: Positron emission tomography, ERCP: encoscopic retrograde cholangio-pancreatography, MRCP: magnetic resonance cholangiopancreatography. Journal title abbreviations conform to the ISO 4 standards.
