# Supplementary material for: Case report: Von Hippel-Lindau syndrome with multisystem involvement: a therapeutic dilemma
Source: Front Oncol. 2025 Oct 14;15:1633911. doi: 10.3389/fonc.2025.1633911 (PMC12558800; doi:10.3389/fonc.2025.1633911)
Supplement: Supplementary file 4 [file DataSheet1.docx]

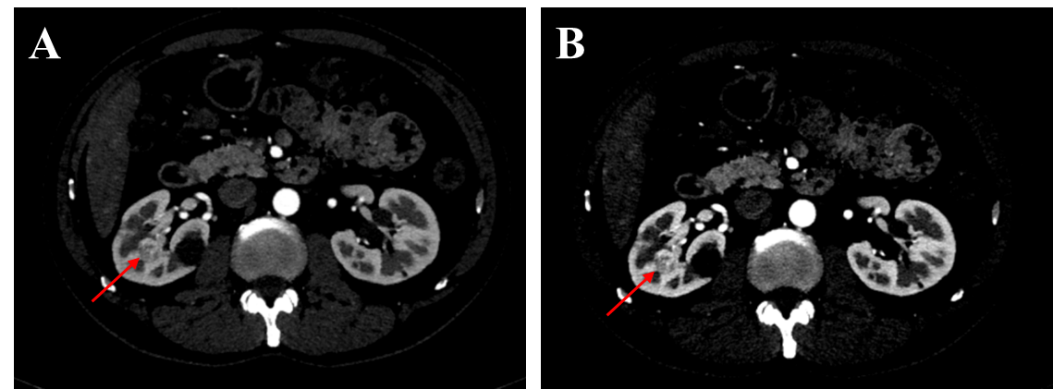


**Supplementary Figure.1 Contrast-enhanced CT imaging of the left kidney with optimized window settings.** (A-B) Axial contrast-enhanced CT image acquired with adjusted window width/level, demonstrating a well-defined hypodense nodule (arrow) measuring 1.30 × 1.40 cm in the renal parenchyma.


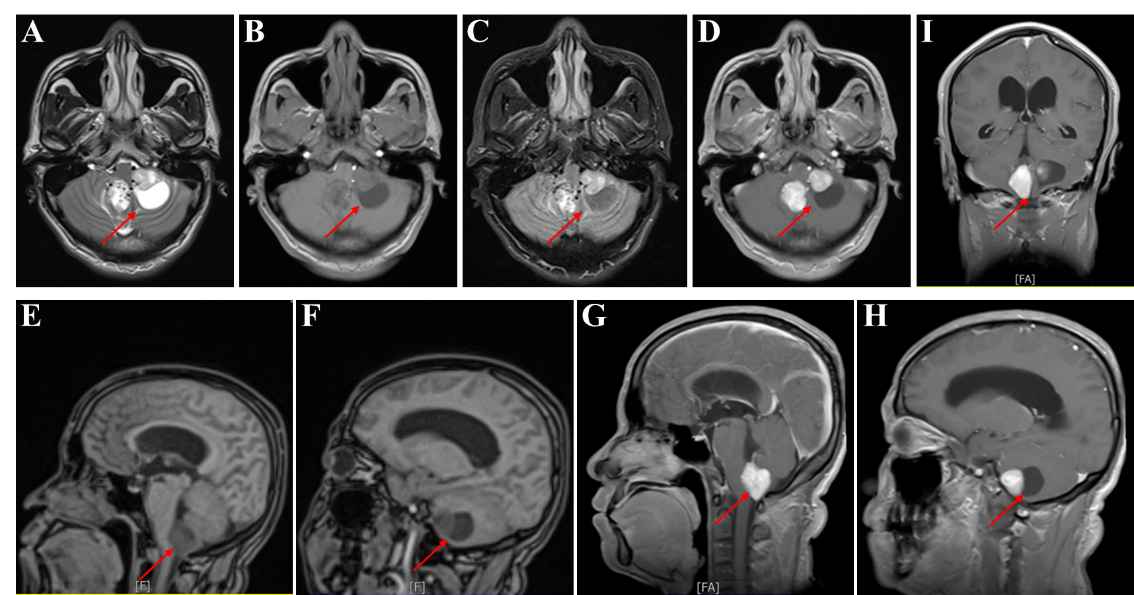


**Supplementary Figure.2 The MRI findings of the patient.**

**
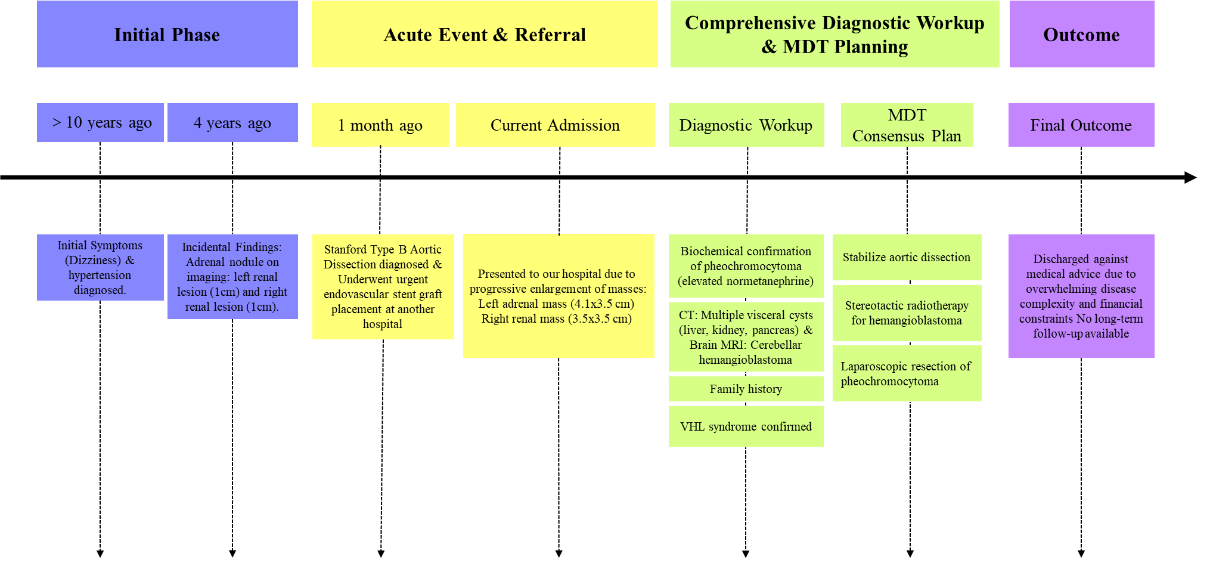
**

**Supplementary Figure.3 The clinical timeline of the presented case with von Hippel-Lindau (VHL) syndrome.** MDT, multidisciplinary team.


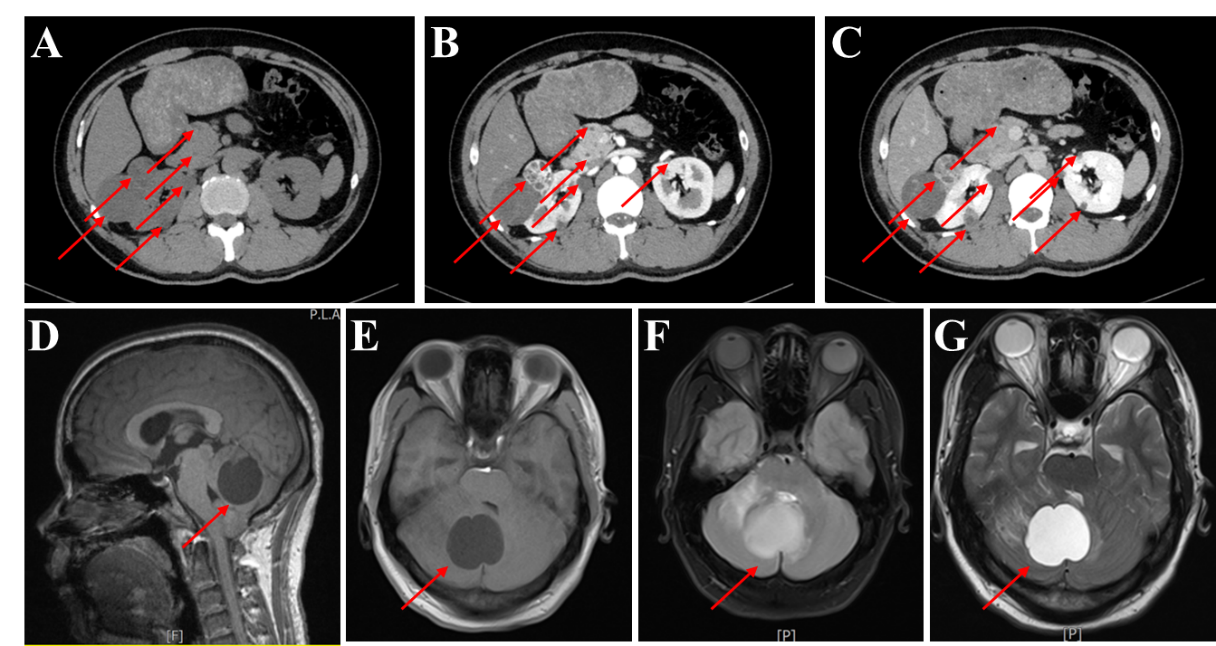


**Supplementary Figure.4 The findings of contrast-enhanced upper abdominal CT and brain magnetic resonance imaging in a VHL patient.** (A-C) Diagnostic contrast-enhanced abdominal CT at VHL syndrome confirmation (2024). (D-G) Brain magnetic resonance imaging (MRI) obtained seven years prior to confirmation of VHL syndrome confirmation (2017).

**
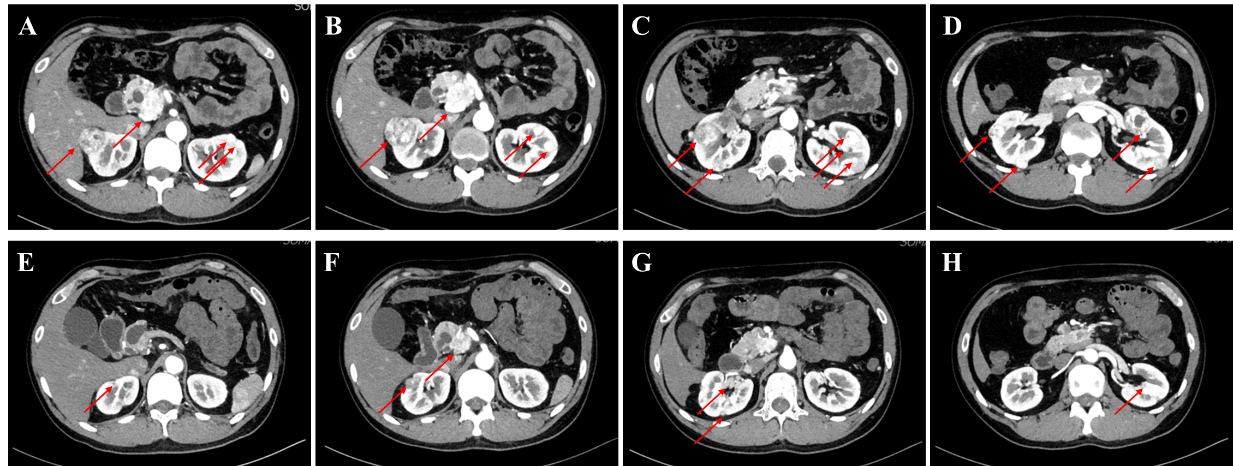
**

**Supplementary Figure.5 Longitudinal contrast-enhanced CT evaluation of upper abdominal lesions before and after anlotinib therapy.** (A-D) Baseline imaging prior to targeted therapy (2021). (E-H) Post-treatment imaging after 36-month anlotinib regimen (2024).
